# Supplementary material for: Assessing concentration in the monoclonal antibody innovation market: A patent-based study
Source: PLoS One. 2025 Mar 27;20(3):e0320864. doi: 10.1371/journal.pone.0320864 (PMC11949330; doi:10.1371/journal.pone.0320864)
Supplement: S1 Table — (DOCX) [file pone.0320864.s001.docx]

# Table S1. Grouping of companies for analyzing monoclonal antibodies patent holders

| Group | Company |
| --- | --- |
| Abbott | Abbott Biotech LTD  Abbott Biotech LTD Bermuda  Abbott Biotherapeutics Corp  Abbott GMBH & CO KG  Abbott Lab S A  Facet Biotech Corp |
| Abbvie | Abbvie Biotechnology LTD  Abbvie Biotechnology LTD  Abbvie Biotherapeutics INC  Abbvie Deutschland GMBH & CO KG  Abbvie INC |
| Alcafleu | Alcafleu Man GMBH & CO KG |
| Amgen | Abgenix Inc  Amgen Inc  Amgen Fremont Inc  Amgen K A Inc  Immunex Corp  Micromet AG |
| Amneal | Amneal Pharmaceuticals LLC |
| Apeiron | Apeiron Biolog AG |
| AstraZeneca | Alexion Pharma INC  Astrazeneca AB  Medimmune Inc  Medimmune LTD  Medimmune LLC  Medlmmune LLC |
| Aurobindo | Acrotech Biopharma LLC |
| Basf | Basf AG |
| Basf | Basf AG |
| Bausch | Valeant Pharmaceuticals Luxembourg S.`a.r.l. |
| Baxter | Baxter AG |
| Bayer | Bayer AG |
| Biogen | Biogen Idec Inc  Biogen Inc A Massachusetts Cor  Biogen Inc  Biogen MA Inc  Idec harmaceuticals  Idec Pharma Corp |
| Bioventure | Bioventure Investments KFT |
| Biovitrum | Arexis AB  Swedish Orphan Biovitrum AB (publ) |
| BMS | Bristol Myers Squibb Co  E R Squibb & Sons L L C  Medarex Inc  Medarex L L C  Squibb Bristol Myers Co  Squibb & Sons Llc |
| Boehringer | Boehringer Ingelheim Pharma  Boehringer Ingelheim Int  Boehringer Igelheim Internat Gmbh  Boehringer Ingelheim Pharmaceuticals, Inc. |
| Boston Scientific | British Tech Group |
| Cambridge Antibody | Cambridge Antibody Tech  Cambridge Antibody Techonology Limited |
| Celltech | Celltech R&D Ltd  Celltech Ltd  Celltech Therapeutics Ltd |
| Celltrion | Celltrion, Inc. |
| Coherus | Coherus Biosciences Inc |
| Cyanamid | American Cyanamid Co |
| Daiichi Sankyo | Daiichi Sankyo Co Ltd  Sankyo Co |
| Elan | Athena Neurosciences Inc  Elan Pharaceuticals Inc |
| Emergent | Emergent Manufacturing Operations Baltimore LLC |
| Fresenius Kabi | Fresenius Kabi USA, LLC |
| Genmab | Genmab As |
| GSK | Burroughs Wellcome Co  Glaxo Wellcome Inc  Glaxosmithkline Llc  Human Genome Sciences Inc  Smithkline Beecham Plc  Smithkline Beecham Corp |
| JT | Japan Tobacco Inc |
| Johnson & Johnson (J&J) | Cantocor Inc  Centocor Inc  Centocor Ortho Biotech Inc  Janssen Biotech Inc |
| Kirin | Kyowa Hakko Kirin Co Ltd  Kyowa Hakko Kogyo KK |
| Lilly | Eli Lilly and Company  Immunogen Inc  Imclone Llc  Lilly Co Eli |
| Lowy | Lowy Israel |
| Meridian | Meridian Medical Technologies |
| Morphosys | Morphosys Ag  Morpho Sys Ag |
| MSD | Merck Patent Gmbh  Merck Sharp & Dohme  Merck Sharpe & Dohme B V  Schering Corp |
| NightHawk | Elusys Therapeutics, Inc. |
| Novartis | Cetus Corp  Cetus Oncology Corp  Chiron Corp  Novartis Ag  Novartis Pharma Gmbh  Novartis Vaccines & Diagnostic  Novarts Ag  Sandoz Inc. |
| OMRF | Oklahoma Med Res Found |
| Ono | Ono Pharmaceutical Co |
| PDL | Pdl Biopharma Inc  Protein Design Labs Inc |
| Peptech | Peptech Ltd |
| Peptide Technology | Peptide Technology Ltd |
| Pfizer | Pfizer Inc. |
| Phigenix | Phigenix Inc |
| Recordati | EUSA Pharma (UK) Limited |
| Regeneron | Regeneron Pharma  Regeneron Pharmacueuticals Inc |
| Roche | Chugai Pharmaceutical Co Ltd  Chugai Seiyaku Kabushika Kaisha  Genentech Inc  Glycart Biotechnology Ag  Hoffmann La Roche  Roche Glycart Ag |
| Royalties | Pharmaceutical royalties l l c |
| Samsung | Samsung Bioepis Co., Ltd. |
| Sanofi | Aventis Pharm Prod Inc  Hoechst AG  Genzyme Corp  Rhone Poulenc Rorer Pharma  Sanofi Biotechnology  Sanofi AS |
| Scotgen | Scotgen Biopharmaceuticals Inc |
| Seagen | Seagen Inc.  Seattle Genetics Inc |
| Senoro | EMD Serono, Inc. |
| Spectrum Pharmaceuticals | Rit Oncology, LLC |
| Takeda | Millennium Pharm Inc  Takeda Pharmaceuticals U.S.A., Inc. |
| Tanox | Tanox Biosystems Inc |
| Teva | Teva Respiratory, LLC |
| UCB | UCB Biopharma Sprl  UCB Celltech  UCB Inc  UCB Pharma AS |
| United | United Therapeutics Corporation |
| Weizmann Institute of Science | Yeda Res & Dev |
| Wyeth | Genetics Inst  Inst Genetics Llc  Wyeth Corp  Wyeth Holdings Llc |
| Xoma | Xoma Technology Ltd  Xoma Corp  Xoma Ltd |
