# Supplementary material for: Assessing concentration in the monoclonal antibody innovation market: A patent-based study
Source: PLoS One. 2025 Mar 27;20(3):e0320864. doi: 10.1371/journal.pone.0320864 (PMC11949330; doi:10.1371/journal.pone.0320864)
Supplement: S2 Table — (DOCX) [file pone.0320864.s002.docx]

# Table S2. List of indicators used in the analysis and their economic relevance

| Group | Indicator | Description |
| --- | --- | --- |
| Concentration | Market concentration | Empirical work has historically used concentration as a relevant structural failure related to anticompetitive behavior. The most used indicators used for concentration are the Herfindahl-Hirschman Index (HHI) and the Concentration Ratio (CR). In this work, the HHI, CR_10_, and CR_4_ were used. The HHI and CR values for patents indicate the market reserve around the world. The HHI and CRs for priorities indicate the concentration on technology development. |
| Strategies to wield market  Power | Participation of different holder types in technology  development (η). | The participation ratio η was calculated for each holder (number of priorities divided by the number of patents). Holders that focus on innovation tend to have η close to one. Patent holders who spread patents tend to have η close to zero. With a kernel graph divided by holder types, it was possible to determine which types of holders focus more on knowledge creation and which focus more on market protection. |
|  | Delay in family spread | This indicator aims to evaluate the rhythm of market protection. The fast spread of a patent family indicates the high formation of a market reserve. In addition, a family registered in only a few countries indicates that the technological capacity to replicate technology is not widespread. |
| Interdependence  among holders | Patent holder partnerships in R&D | The indicator was calculated by aggregating the number of holders per patent and priority. This indicator refers to the dependency of different players on the process of knowledge creation. This dependency reflects on the number of players that are included in the licensing process of each patent. The licensing process impacts on the innovation market because more players are associated with the bureaucratization of the approval of derived patents and the division of royalties. The greater the dependency between players, the less autonomy companies have to explore the protected knowledge. |
|  | Company centrality in molecule development | A network linking molecules and priority patent holders was built. Since a molecule might be associated with multiple priorities, this network shows the most relevant holders. Holders with a higher degree of centrality are responsible for important patents in the process of development of a new drug; i.e., the other companies depend on them for the chain of knowledge generation. The greater the interdependency between players, the less autonomy companies have to explore the knowledge |
