# Supplementary material for: Assessing concentration in the monoclonal antibody innovation market: A patent-based study
Source: PLoS One. 2025 Mar 27;20(3):e0320864. doi: 10.1371/journal.pone.0320864 (PMC11949330; doi:10.1371/journal.pone.0320864)
Supplement: S3 Table — GNPO = Government Non-Profit Organization; NGO = Non-Government Organization. (DOCX) [file pone.0320864.s003.docx]

# Table S3. Patent and priority holders by type and partnerships

| Holders | mAb Patents | mAb Priorities | Chem patents | Chem priorities |
| --- | --- | --- | --- | --- |
| One company | 808 | 211 | 2,024 | 504 |
| One individual | 64 | 28 | 197 | 38 |
| One GNPO | 24 | 16 | 11 | 3 |
| One hospital | 20 | 15 | 1 | 1 |
| One university | 35 | 21 | 85 | 10 |
| One NGO | 0 | 0 | 0 | 0 |
| One unknown | 0 | 0 | 6 | 2 |
| Multiple companies | 96 | 14 | 316 | 43 |
| Multiple individuals | 136 | 40 | 337 | 54 |
| Multiple GNPOs | 0 | 0 | 0 | 0 |
| Multiple Hospitals | 0 | 0 | 0 | 0 |
| Multiple universities | 36 | 30 | 8 | 7 |
| Multiple NGOs | 0 | 0 | 0 | 0 |
| Multiple unknown | 0 | 0 | 0 | 0 |
| Company + individual + university + hospital | 0 | 0 | 1 | 0 |
| Company + GNPOs + individuals | 7 | 0 | 1 | 0 |
| Company + individuals + universities | 6 | 1 | 13 | 0 |
| Hospital + individuals + universities | 0 | 0 | 1 | 0 |
| GNPOs + NGOs + university | 0 | 0 | 1 | 0 |
| Companies + individuals | 394 | 35 | 1,515 | 155 |
| Companies + GNPOs | 24 | 4 | 2 | 0 |
| Companies + universities | 11 | 9 | 24 | 2 |
| GNPOs + individuals | 11 | 9 | 6 | 0 |
| GNPOs + universities | 5 | 5 | 4 | 1 |
| NGOs + company | 0 | 0 | 2 | 0 |
| Individuals + universities | 22 | 5 | 42 | 4 |
| Hospital + individuals | 4 | 2 | 1 | 0 |
| Companies + unknown | 0 | 0 | 7 | 2 |
| Individuals + unknown | 0 | 0 | 3 | 1 |
| Total | 1,703 | 445 | 4,608 | 827 |

GNPO = Government Non-Profit Organization; NGO = Non-Government Organization.
