# Supplementary figures and images for: Assessing concentration in the monoclonal antibody innovation market: A patent-based study
Source: PLoS One. 2025 Mar 27;20(3):e0320864. doi: 10.1371/journal.pone.0320864 (PMC11949330; doi:10.1371/journal.pone.0320864)

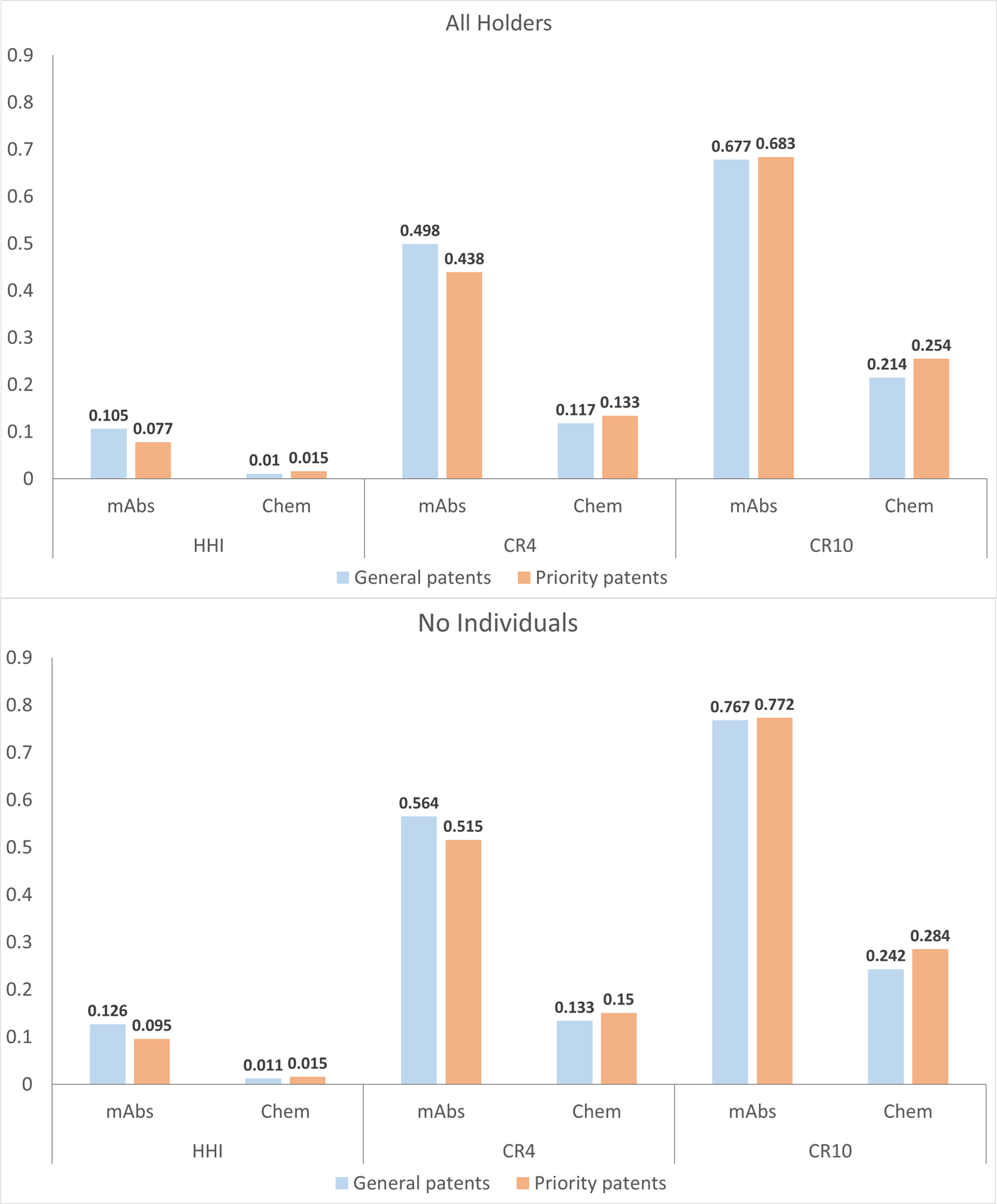

Supplement: S4 Fig — HHI = Herfindahl-Hirschman Index; CR = Concentration Ratio (TIF) [file pone.0320864.s004.tif]

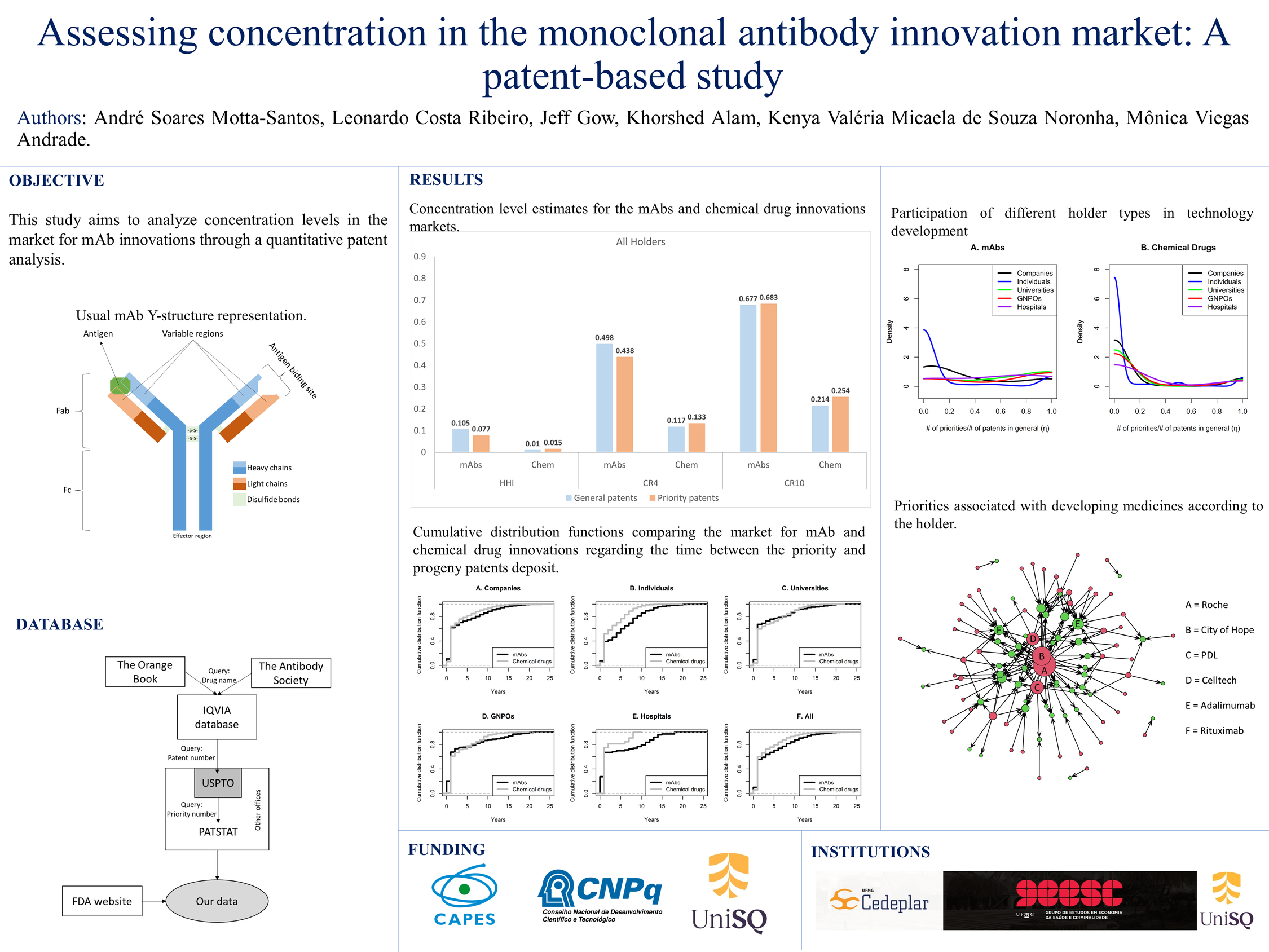

Supplement: S6 Fig — (TIF) [file pone.0320864.s006.tif]
