# Supplementary material for: Assessing concentration in the monoclonal antibody innovation market: A patent-based study
Source: PLoS One. 2025 Mar 27;20(3):e0320864. doi: 10.1371/journal.pone.0320864 (PMC11949330; doi:10.1371/journal.pone.0320864)
Supplement: S5 Table — mAb associated priorities and their indications # = Number; US = United States of America (DOCX) [file pone.0320864.s005.docx]

# Table S5. mAb associated priorities and their indications

| # | # of referring drugs | # of indications | Country | Filling year | # of holders | Type of holders | # of holders per type |
| --- | --- | --- | --- | --- | --- | --- | --- |
| 1 | 24 | 112 | US | 1988 | 1 | Company | 1 |
| 2 | 23 | 111 | US | 1995 | 7 | Company Individual University | 1 5 1 |
| 3 | 15 | 68 | US | 2003 | 1 | Company | 1 |
| 4 | 15 | 68 | US | 2003 | 1 | Company | 1 |
| 5 | 15 | 68 | US | 1995 | 1 | Company | 1 |
| 6 | 15 | 64 | US | 1983 | 1 | Company | 1 |
| 7 | 15 | 68 | US | 1995 | 1 | Company | 1 |
| 8 | 15 | 68 | US | 1995 | 1 | Company | 1 |
| 9 | 15 | 68 | US | 2000 | 1 | Company | 1 |
| 10 | 14 | 63 | US | 1995 | 1 | Company | 1 |

# = Number; US = United States of America
