# Supplementary material for: Assessing concentration in the monoclonal antibody innovation market: A patent-based study
Source: PLoS One. 2025 Mar 27;20(3):e0320864. doi: 10.1371/journal.pone.0320864 (PMC11949330; doi:10.1371/journal.pone.0320864)
Supplement: Attachment — Submitted filename: Comments.pdf [file pone.0320864.s007.pdf]

## Assessing Concentration in the Monoclonal Antibody Innovation Market: A Patent- Based Study

Motta-Santos and colleagues studied the thorough analysis of mAb Market and its implications for healthcare innovation. They highlighted the concentration levels and dissemination patterns of mAb patents

Authors can improve the quality of the manuscript by considering following modifications:

- In the Introduction, authors can highlight the research's novelty and its effectiveness for stakeholders.
- Authors can also discuss the impact of biosimilars on the mAb market.
- Authors can also add some of the case studies or specific examples of successful R&D projects that led to significant changes in mAbs developments or markets.
- Lane 135: Authors can include specific examples of how geographic boundaries influence market dynamics, such as regional variations in drug availability and pricing.
- Authors can discuss about retrieving data for chemical drug patents parallels the process for mAbs with the explanation of major differences.
- Please explain the use of the Herfindahl-Hirschman Index (HHI) and Concentration Ratio (CR). Please add a brief discussion on the significance of these indices in other industries so that readers who are unfamiliar with these concepts understand their relevance.
- The authors provided plenty of data in the table format and text explanation. Authors can consider making bar graphs for the results shown in the table to represent the distribution of patents among holder types for an easy and thorough understanding of the readers. For market concentration, add graphical representations of HHI and CR values.
- Line 358-365: Please explain why is the decrease in participation significant for market dynamics.
- Lines 406–439: Please explain why single-holder contributions dominate and the effect on innovation dynamics. Please explain the barriers to cross-holder partnerships if there are any.
- In Figures 2&3: Please explain why the slope difference matters for market concentration or innovation strategies. What do slopes mean for policy or market behavior?
