# Supplementary material for: Assessing concentration in the monoclonal antibody innovation market: A patent-based study
Source: PLoS One. 2025 Mar 27;20(3):e0320864. doi: 10.1371/journal.pone.0320864 (PMC11949330; doi:10.1371/journal.pone.0320864)
Supplement: Attachment — Submitted filename: Response to Reviewers.docx [file pone.0320864.s008.docx]

**PONE-D-24-54902**

Assessing Concentration in the Monoclonal Antibody Innovation Market: A Patent-Based Study

Please submit your revised manuscript by Feb 19 2025 11:59PM. If you will need more time than this to complete your revisions, please reply to this message or contact the journal office at plosone@plos.org.

Please include the following items when submitting your revised manuscript:

1. A rebuttal letter that responds to each point raised by the academic editor and reviewer(s). You should upload this letter as a separate file labeled 'Response to Reviewers'.
2. A marked-up copy of your manuscript that highlights changes made to the original version. You should upload this as a separate file labeled 'Revised Manuscript with Track Changes'.
3. An unmarked version of your revised paper without tracked changes. You should upload this as a separate file labeled 'Manuscript'.
4. If you would like to make changes to your financial disclosure, please include your updated statement in your cover letter. Guidelines for resubmitting your figure files are available below the reviewer comments at the end of this letter.

Dear reviewers,

We would like to thank you for your careful read of our article and your comments. We truly believe that they help us improve our paper. We addressed all issues appointed by you and provided answers to your questions below.

Best regard,

The authors

# REVIEWERS' COMMENTS

# Reviewer 1

## Comment 1. Abstract should be formatted objective/method/results/conclusion.

A: Thanks for this correction. The abstract was re-structured as requested. Please, find the modified version below.

**BACKGROUND**: Monoclonal antibodies (mAbs) are revolutionizing healthcare treatments due to their high efficacy and relative safety, despite their cost. Since they first appeared in the late 1980s, a rapidly growing market has developed. **OBJECTIVE**: This study aims to analyze concentration levels in the market for mAb innovations through a quantitative patent analysis. Data were analyzed using traditional concentration indicators such as the Herfindahl-Hirschman Index and Concentration Ratio, as well as linear regression and kernel density graphs to evaluate innovation and global technology dissemination strategies. The starting point was patents associated with mAbs registered by the FDA and identified in the IQVIA database up until 2019, supplemented by data from The Antibody Society, Purple Book, Orange Book, and FDA. **RESULTS**: Our findings indicate that the market for mAb innovations is moderately concentrated for general patents and unconcentrated for priority patents. However, it is significantly more concentrated than the market for chemical drug innovations. The mAb patent families tend to generate more progeny patents, although they are deposited in fewer countries. Chemical drug patents spread faster. Some companies seem to be central to the development of mAbs worldwide, including Roche, PDL, City of Hope, and Celltech. Other important players in the mAb innovation market are AbbVie, Amgen, Novartis, GSK, Biogen, BMS, Regeneron, J&J, and AstraZeneca. The most relevant patents in the analysis are associated with methods and procedures to obtain mAbs, not with molecules themselves. **CONCLUSION**: The concentration in the mAb innovation market is higher than the concentration in the market for chemical drugs innovations. Our findings also indicate that expertise in mAbs development and production is concentrated in a few countries. Additionally, our study identified that a few key players from high-income countries are driving innovation in the mAb market.

## Comment 2. Introduction should touch 360 angle of topic.

A: Thank you for this comment. To address it, we included some additional information in the introduction. They include the development of second generation mAb technology and their future potential:

Recently, the development of antibody-drug conjugates (ADCs) and bispecific antibodies has widened even more the usefulness of mAb technology, especially for cancer treatments [17–22]. In the short term, continued investment in R&D for new mAb technologies could provide the world with technologies to treat or cure many rare and orphan diseases [5,6,8,23,24].

A brief history of successful research associated with mAbs and their economic importance for healthcare:

The onset of mAb research happened in 1975, when Milstein and Köhler [25] described a method for obtaining specific antibodies from a continuously growing cell line. In 1986, after some additional advances, the first monoclonal antibody (mAb), Muromonab-CD3, was approved for human use by the Food and Drugs Administration (FDA) [7,14]. However, it was only after the development of the methods for obtaining chimeric, humanized, and human mAbs that the new molecules start getting in the market at an increasing pace [4,26,27]. mAbs are associated with substantial research and development (R&D) costs. According to DiMasi and Grabowski [28], the average capital cost to develop a new biopharmaceutical drug was approximately US$1.2 billion during the 2010s. Despite these significant expenses, mAbs high prices consistently deliver translate into abundant revenues for pharmaceutical companies, making them a lucrative investment [29–31].

An estimates for the concentration on the production market and our focus, that is, the concentration in the innovation market. The last paragraph now reads:

In the pharmaceutical market, three related processes – R&D, production, and commercialization – could be carried out by different companies for the same product, leading to three different estimates of market concentration. Specifically, for the drug production market, there is some evidence on concentration levels [49,50]. They observe that the market as a whole is not concentrated enough to be considered an oligopoly. Craig and Malek [49] found that the five largest pharmaceutical companies retained around 15% of the market in the 1990s [49]. Information on the innovation market, especially mAbs, is much scarcer. This study focuses on R&D by analyzing patent deposit activities, with the specific aim of measuring concentration in the mAbs innovation market using an unprecedent database. Understanding the interactions among players and concentration in the innovation market is essential for developing policies to address the current high prices of mAbs. In addition, the dynamism of the field requires up-to-date analyses. To the best of our knowledge, this study is the first comprehensive analysis of mAbs innovation market.

Some smaller modifications were also introduced to make the introduction clearer and more precise.

## Comment 3. Conclusion need to rewrite for effective delivery of research analysis.

A: We modified and removed parts of the conclusion to make it more concise and to the point. Please see the result below:

The main contribution of this paper is the analysis of the concentration within the pharmaceutical innovation market, using a dedicated database to examine the strategies companies employ to maintain market power. Our findings also indicate that expertise in mAbs development and production is concentrated in a few countries, leaving many regions without the necessary capabilities to innovate or manufacture mAbs. Additionally, our study identified key players driving innovation in the mAb market: Roche, PDL, City of Hope, and Celltech. Other significant contributors include AbbVie, Amgen, Novartis, GSK, Biogen, BMS, Regeneron, J&J, and AstraZeneca.

# Reviewer 2 - PALLAPATI ANUSHA RANI

## Comment 1. In the Introduction, authors can highlight the research's novelty and its effectiveness for stakeholders.

A: Hi. Thank you for this opportunity to include other information to demonstrate the importance of our paper. We included modifications in the last paragraph of the introduction. It now reads:

In the pharmaceutical market, three related processes – R&D, production, and commercialization – could be carried out by different companies for the same product, leading to three different estimates of market concentration. Specifically, for the drug production market, there is some evidence on concentration levels [49,50]. They observe that the market as a whole is not concentrated enough to be considered an oligopoly. Craig and Malek [49] found that the five largest pharmaceutical companies retained around 15% of the market in the 1990s [49]. Information on the innovation market, especially mAbs, is much scarcer. This study focuses on R&D by analyzing patent deposit activities, with the specific aim of measuring concentration in the mAbs innovation market using an unprecedent database. Understanding the interactions among players and concentration in the innovation market is essential for developing policies to address the current high prices of mAbs. In addition, the dynamism of the field requires up-to-date analyses. To the best of our knowledge, this study is the first comprehensive analysis of mAbs innovation market.

## Comment 2. Authors can also discuss the impact of biosimilars on the mAb market.

A: Included in the discussion, as requested. Please, see the new paragraph below.

Despite the complications of copying mAbs, the market for biosimilars have been gaining traction over the years. Biological drugs are much more difficult to imitate than chemical drugs. From an economic perspective, the production of biosimilars involves more barriers to entry than the production of small molecules [80]. Specifically, in the case of mAbs, there is an essential differentiation of products. Biosimilars are not generic mAbs. They can be different with respect to the protein sequence and, thus, effects. The expiration of many mAb patents in the next few years should ignite the development of biosimilars. Remicade® (infliximab) sales were slowed by 15% due to biosimilars between 2015 and 2017. A drop that is still modest, considering the prices of reference mAbs. Developing biosimilars that act better than reference products, also known as biobetters or biosuperiors, should accelerate this process [12] and help reduce the concentration in the market for production of mAbs. However, their imitative nature suggests they will not affect innovation market concentration.

## Comment 3. Authors can also add some of the case studies or specific examples of successful R&D projects that led to significant changes in mAbs developments or markets.

A: We feel that a good example of a successful R&D project is the Cabilly patents described in the discussion. However, to better comply with this comment, we included a paragraph describing some important research results that helped launched mAbs in the introduction:

The onset of mAb research happened in 1975, when Milstein and Köhler [25] described a method for obtaining specific antibodies from a continuously growing cell line. In 1986, after some additional advances, the first monoclonal antibody (mAb), Muromonab-CD3, was approved for human use by the Food and Drugs Administration (FDA) [7,14]. However, it was only after the development of the methods for obtaining chimeric, humanized, and human mAbs that the new molecules start getting in the market at an increasing pace [4,26,27]. mAbs are associated with substantial research and development (R&D) costs. According to DiMasi and Grabowski [28], the average capital cost to develop a new biopharmaceutical drug was approximately US$1.2 billion during the 2010s. Despite these significant expenses, mAbs high prices consistently deliver translate into abundant revenues for pharmaceutical companies, making them a lucrative investment [29–31].

## Comment 4. Lane 135: Authors can include specific examples of how geographic boundaries influence market dynamics, such as regional variations in drug availability and pricing.

A: We appreciate this comment and included a more robust explanation of the geographic boundaries in the pharmaceutical market. Please, find the modification below:

Markets must be defined with respect to their geographic and product dimensions. The geographic dimension comprises the territorial boundaries of the market; i.e. all relevant sources of the product and their spatial disposition. Since the formation of large drug companies in the last century, the borders of pharmaceutical markets have expanded [49]. Despite some trade restrictions between countries imposed by the TRIPS Agreement, the pharmaceutical production market has a global scope in the sense that commercialization of drugs might happen across frontiers [33,52,53]. The same is true for the innovation market. The flow of information occurs across borders through the allocation and enforcement of intellectual property rights (IPRs) in offices in each country. However, patent legislation per se are contained inside the borders of national innovation systems. The dependency of international products subjected to IPR protection presents two challenges for the access to new drugs, especially in LMICs: (i) the monopoly created by the IPR hinders competition and lead to higher prices and (ii) the logistics of distributing a new technology around the world is difficult. Even registering new technologies with local authorities might delay the availability of new drugs.

## Comment 5. Authors can discuss about retrieving data for chemical drug patents parallels the process for mAbs with the explanation of major differences.

A: This is a good idea. Please, find the paragraph re-written below:

Figure 1 synthesizes the steps followed to build the database. The first step is the identification of the drugs. The list of mAbs approved by the FDA and their International Non-Proprietary Names (INNs) were collected from The Antibody Society website [57–59]. The Antibody Society is an international non-profit trade association representing individuals and organizations involved in antibody development. The list of INNs of chemical drugs approved by the FDA was collected from the Orange Book [60]. The Orange Book (Approved Drug Products with Therapeutic Equivalence Evaluations) identifies drug products approved by the FDA under the Federal Food, Drug, and Cosmetic Act. The INNs were used to retrieve the US Patent and Trademark Office (USPTO) patent numbers from the IQVIA database, which include data from 130 countries [61].

## Comment 6. Please explain the use of the Herfindahl-Hirschman Index (HHI) and Concentration Ratio (CR). Please add a brief discussion on the significance of these indices in other industries so that readers who are unfamiliar with these concepts understand their relevance.

A: To address this issue, two parts of the text were changed. Initially, the significance of the indices were better explained in the methods section:

This paper applies the HHI and the CR (CR4 and CR10) to estimate market concentration. These indices are among the most well-established in the literature. Generally, they evaluate the distribution of market share, measured in our study by patents, among individuals or firms. Therefore, they estimate the competition in an industry [68]. The CR is the cumulative market share of the M largest companies in the market (Equation 1) while the HHI is calculated by summing the squares of the market shares of all holders (Equation 2). Their values range from 0 to 1 (monopoly); more precisely, D_(HHI|CR)=(0,1]. A market can be considered unconcentrated when HHI≤0.10, moderately concentrated between 0.10 and 0.18, and highly concentrated when HHI>0.18 [69–72]. For I companies on a market, the HHI has its lowest value when all players have equal market shares; that is, min(HHI)=1⁄I [68,71]. In the case of the CR, an industry is commonly considered competitive when CR_4<0.4 while CR_4>0.6 is associated with tight oligopolies or a market with a dominant firm with a competitive fringe [68].

A new paragraph was added to the discussion to address this comment as well.

It is paramount to understand how the HHI and CR relate to each other. The relationship between them is not monotonic and depends on the market being evaluated [68]. They do not necessarily indicate the same result, but the analysis of both can lead to some conclusions. One inference that can be drawn from our concentration indices is that the mAb innovation market comprises a core of large companies surrounded by numerous smaller firms; i.e. an oligopoly with a competitive fringe. The high concentration in the mAb innovation market is a significant factor contributing to elevated drug prices. Estimates of HHI and CR4 for different industries are numerous. Each industry has its own specificities and trends. For comparison, some of examples are: the US consumer book industry (HHI=0.118, CR4=0.368) [77], the global personal computers industry (HHI=0.148, CR4=0.707) [78], the Turkish automobile industry (HHI=0.079, CR4=0.462), and the biodiesel industry in Brazil (HHI=0.059, CR4=0.409) [79].

This should be enough for readers unfamiliar with the use of such indices to understand their meaning.

## Comment 7. The authors provided plenty of data in the table format and text explanation. Authors can consider making bar graphs for the results shown in the table to represent the distribution of patents among holder types for an easy and thorough understanding of the readers. For market concentration, add graphical representations of HHI and CR values.

A: We included S4 Fig. It is a figure with a graphical representation of the data in Table 1. The new figure is presented below.

## Comment 8. Line 358-365: Please explain why is the decrease in participation significant for market dynamics.

A: To address this comment, we included a simple, but easily understandable sentence in the same paragraph. Now it reads:

As explained above, these coefficients represent the elasticities of each estimate. Therefore, the negative signs mean that the increase in the number of holders is associated with a lower participation of holders in general patents and priorities, which is a logical conclusion; i.e., the larger the number of holders, the more competitive (or less concentrated) the market is. A 1% increase in the number of holders is associated with a 0.94% decrease in participation for general mAb patents, a 0.90% decrease for mAb priority patents, a 0.72% decrease for general chemical drug general patents, and a 0.65% decrease for chemical drug priorities. In summary, the market for mAb innovations is more concentrated and shows greater responsiveness to changes in the number of patent holders compared to the market for chemical drug innovations.

## Comment 9. Lines 406–439: Please explain why single-holder contributions dominate and the effect on innovation dynamics. Please explain the barriers to cross-holder partnerships if there are any.

A: Thank you for the opportunity to address this issue. We do not believe there is a barrier to cross-holder partnerships per se. Developing technology as a single holder has some conveniences, such as ease of licensing and decision-making. To comply with this comment, we included this description in the results:

Partnerships might make licensing and agreements between companies more complex and reduce gain from royalties, which would incentivize players to avoid them when possible.

## Comment 10. In Figures 2&3: Please explain why the slope difference matters for market concentration or innovation strategies. What do slopes mean for policy or market behavior?

A: Thank you for the opportunity to clarify this issue. As we stated in the methods section – Market Concentration – “Linear regression coefficients are associated with the degree of concentration. Higher absolute values of the coefficient (i.e., the slope of the log-log regression curve) indicate more concentrated markets.” But we understand that non-economists might have difficulty interpreting this result. Therefore, we made a change to the first paragraph of the discussion to include that:

Higher concentration is associated with less competitive markets and, consequently, higher prices.

# Reviewer 3

## Comment 1. Lack of correlation between patent concentration and market share: The paper focuses on patent concentration (HHI based on patents) but fails to connect this to actual market share of the corresponding mAb products. A crucial aspect is understanding whether the companies with the highest patent concentration also hold the largest market share of approved and marketed mAb drugs. This would provide a more direct link between patent dominance and potential market power.

A: Thank you for this comment. We would really like to have the answer for this. However, the most important patents in the pharmaceutical market are not related to products, but intermediary processes in the way to obtain them. A single drug might have been associated with dozens of patents. The average number of patents associated with each mAb was 41.90 (s=61.14, min=1, median=21, max=387) from diverse holders. The construction of healthcare technology requires multidisciplinary knowledge. Therefore, the relationship between patent concentration and market share in the production market is not direct. There are even companies that hold important patents and do not produce a single molecule. Please, notice that we tried to show the most important players through network analysis and the most important patents through the number of molecules they were paramount to develop (Figure 6 and Table S5). Those players are the ones that have been most instrumental to the development of new technology.

Network analysis shows that Roche Group, City of Hope, PDL, and Celltech are the most relevant institutions in the development of new mAbs and the intermediary processes to produce them (high-degree centrality in the directed network). The degree centrality quantifies the power of the node [82], i.e. how well connected it is. Therefore, these companies hold patents that are generally more important for the mAb market. The number of molecules associated with a priority indicates its usefulness and application. Companies with priority patents associated with more drugs tend to have more influence over the market.

We also look the literature to find the most important players in the mAb market and, as expected, they are among the most important in the patent network.

The leading players in the mAb innovation market align closely with the dominant companies in the production market. Notably, several companies holding the highest number of patents (Roche, AbbVie, Amgen, Novartis, GSK, Biogen, BMS, Regeneron, J&J, and AstraZeneca) have also been recognized as major players in the production market. In 2017, seven companies dominated the mAb market, accounting for 87% of the sales. They were (i) Genentech (Roche Group) with 31% and 11 molecules; (ii) AbbVie with 20.0%; (iii) J&J with 13.6%; (iv) BMS with 6.5%; (v) Merck Sharp & Dohme (MSD) with 5.6%; (vi) Novartis with 5.5%; and (vii) Amgen with 4.9% [12]. One possible explanation for this high correlation among players in both the innovation and production mAb market is patent protection and the advantage of being a first-mover.

To better comply with your suggestion, we amended this limitation to include the information that seem to have produced the issue. It now reads:

The use of HHI or CR for concentration is not informative of the relevance of each invention or market shares. Therefore, a holder with a small number of patents can be more important than a holder with many less relevant patents. The most important patents in the pharmaceutical market are not related to products, but intermediary processes in the way to obtain them. The average number of patents associated with each mAb was ranged from 1 to 387 from diverse holders. Therefore, the relationship between patent concentration and market share in the production market is not direct. There are even companies that hold important patents and do not produce a single molecule. To mitigate this limitation, we conducted network analysis and examined the most frequently cited priorities.

## Comment 2. The paper mentions "moderate" and "unconcentrated" markets, but lacks a deeper analysis of the potential implications of these concentration levels: (i) Potential for anti-competitive behavior: Does the level of concentration raise concerns about potential anti-competitive practices like price-fixing or exclusionary behavior? (ii) Impact on innovation: Does the concentration level stifle innovation by hindering entry of new players and limiting access to technology? (iii) Impact on patient access: Does the concentrated market structure potentially lead to higher drug prices and limited patient access to these life-saving therapies?

A: Another paragraph was included in the discussion to address these comments. In this paragraph, it is stated that:

There are some concerns regarding high prices for medicines and the delayed entry of competitors in the market even more than 30 years after the Trade-Related Aspects of Intellectual Property Rights (TRIPS) Agreement was signed [32,33,40,56,80,84]. The high prices of medicines are derived from a complicated entanglement between entry barriers (such as high entry costs, high technology necessity, licensing, promotion, and patent protection), concentration, and market structures. Some authors state that the high prices of medicines lead to catastrophic family expenses and lack of access [85,86]. However, pharmaceutical companies argue that substantial R&D investments are responsible for the high price of medicines, and reductions might compromise the development of new drugs [80,84]. The use of artificial intelligence and machine learning in the development of new drugs might eventually influence this landscape, leading to higher R&D productivity [87,88].

## Comment 3. The analysis appears to be conducted across the entire mAb market without considering the significant variations within different therapeutic areas (e.g., oncology, autoimmune diseases, infectious diseases). Concentration levels and their implications can vary significantly across these areas. Analyzing concentration within specific therapeutic areas would provide a more nuanced understanding of the competitive landscape.

A: We agree with your consideration on this matter. Thank you for noticing. However, it was not in the scope of this work to evaluate specific sub(market). Please, notice that we included this matter in our limitations section:

The cross-elasticity in the pharmaceutical market is low; i.e., products and patents are not generally substitutes for each other. We opted to divide the market into mAbs and chemical drugs for proximity, but these analyses could have been performed considering different drug classes, fields, indications, or even molecules to find specific concentration estimates. However, the analyses of such subgroups are out of the scope of our work. It is first important to understand the market as a whole before we can discuss specific (sub)markets.

This issue is not exclusive of the pharmaceutical market. To comply with another request, we included some examples of HHI and CR estimates from other markets:

Estimates of HHI and CR4 for different industries are numerous. Each industry has its own specificities and trends. For comparison, some of examples are: the US consumer book industry (HHI=0.118, CR4=0.368) [79], the global personal computers industry (HHI=0.148, CR4=0.707) [80], the Turkish automobile industry (HHI=0.079, CR4=0.462), and the biodiesel industry in Brazil (HHI=0.059, CR4=0.409) [81].

If we take the US consumer book industry, we are subjected to the same limitation. A book is not usually a substitute for another or, in the best case, it is an imperfect substitute. Nevertheless, there is merit in understanding how concentrated this market is. Our focus in this paper is to understand the whole innovation market for mAbs before we can proceed to specific cases which requires a different definition on the product level.

## Comment 4. The paper primarily focuses on past trends and current market conditions. A discussion on potential future trends in the mAb market, such as: (i) The rise of biosimilars and their impact on market concentration. (ii) The increasing importance of emerging technologies like antibody-drug conjugates (ADCs) and bispecific antibodies. (iii) The potential impact of artificial intelligence (AI) and machine learning on mAb development. Would enhance the paper's value and provide insights into the evolving competitive landscape.

A: Thank you for this comment and the opportunity to include such fringe topics in our paper. A paragraph discussing biosimilars was included in the discussion.

Despite the complications of copying mAbs, the market for biosimilars have been gaining traction over the years. Biological drugs are much more difficult to imitate than chemical drugs. From an economic perspective, the production of biosimilars involves more barriers to entry than the production of small molecules [80]. Specifically, in the case of mAbs, there is an essential differentiation of products. Biosimilars are not generic mAbs. They can be different with respect to the protein sequence and, thus, effects. The expiration of many mAb patents in the next few years should ignite the development of biosimilars. Remicade® (infliximab) sales were slowed by 15% due to biosimilars between 2015 and 2017. A drop that is still modest, considering the prices of reference mAbs. Developing biosimilars that act better than reference products, also known as biobetters or biosuperiors, should accelerate this process [12] and help reduce the concentration in the market for production of mAbs. However, their imitative nature suggests they will not affect innovation market concentration.

A comment on ADCs and BsAb was included in the first paragraph of the introduction to acknowledge their importance. It says:

Recently, the development of antibody-drug conjugates (ADCs) and bispecific antibodies has widened even more the usefulness of mAb technology, especially for cancer treatments [17–22].

A comment on the potential influence of AI technology on the R&D productivity was included in the discussion. It says:

The use of artificial intelligence and machine learning in the development of new drugs might eventually influence this landscape, leading to higher R&D productivity [89,90].

We also modified the last paragraph of the introduction to include some more information and make our objective more precise. It reads:

In the pharmaceutical market, three related processes – R&D, production, and commercialization – could be carried out by different companies for the same product, leading to three different estimates of market concentration. Specifically, for the drug production market, there is some evidence on concentration levels [49,50]. They observe that the market as a whole is not concentrated enough to be considered an oligopoly. Craig and Malek [49] found that the five largest pharmaceutical companies retained around 15% of the market in the 1990s [49]. Information on the innovation market, especially mAbs, is much scarcer. This study focuses on R&D by analyzing patent deposit activities, with the specific aim of measuring concentration in the mAbs innovation market using an unprecedent database. Understanding the interactions among players and concentration in the innovation market is essential for developing policies to address the current high prices of mAbs. In addition, the dynamism of the field requires up-to-date analyses. To the best of our knowledge, this study is the first comprehensive analysis of mAbs innovation market.

# Reviewer 4

## Comment 1. Authors should also explore factors such as research and development (R&D) investments, collaborations, and licensing agreements that shape the competitive landscape for the mAb market.

A: Thank you for the comment. We included extra information on these topics. In the introduction, we included another paragraph dealing with R&D in the pharmaceutical market:

The onset of mAb research happened in 1975, when Milstein and Köhler [25] described a method for obtaining specific antibodies from a continuously growing cell line. In 1986, after some additional advances, the first monoclonal antibody (mAb), Muromonab-CD3, was approved for human use by the Food and Drugs Administration (FDA) [7,14]. However, it was only after the development of the methods for obtaining chimeric, humanized, and human mAbs that the new molecules start getting in the market at an increasing pace [4,26,27]. mAbs are associated with substantial research and development (R&D) costs. According to DiMasi and Grabowski [28], the average capital cost to develop a new biopharmaceutical drug was approximately US$1.2 billion during the 2010s. Despite these significant expenses, mAbs high prices consistently deliver translate into abundant revenues for pharmaceutical companies, making them a lucrative investment [29–31].

We also modified the last paragraph of the introduction to include more information on the competitive landscape and to pinpoint our objective more precisely:

In the pharmaceutical market, three related processes – R&D, production, and commercialization – could be carried out by different companies for the same product, leading to three different estimates of market concentration. Specifically, for the drug production market, there is some evidence on concentration levels [49,50]. They observe that the market as a whole is not concentrated enough to be considered an oligopoly. Craig and Malek [49] found that the five largest pharmaceutical companies retained around 15% of the market in the 1990s [49]. Information on the innovation market, especially mAbs, is much scarcer. This study focuses on R&D by analyzing patent deposit activities, with the specific aim of measuring concentration in the mAbs innovation market using an unprecedent database. Understanding the interactions among players and concentration in the innovation market is essential for developing policies to address the current high prices of mAbs. In addition, the dynamism of the field requires up-to-date analyses. To the best of our knowledge, this study is the first comprehensive analysis of mAbs innovation market.

There were other minor modifications during the text that might help address this issue. Thank you for the help.

## Comment 2. Add graphical abstract.

A: That is a great idea. Included.

# PACE

While revising your submission, please upload your figure files to the Preflight Analysis and Conversion Engine (PACE) digital diagnostic tool, https://pacev2.apexcovantage.com/. PACE helps ensure that figures meet PLOS requirements. To use PACE, you must first register as a user. Registration is free. Then, login and navigate to the UPLOAD tab, where you will find detailed instructions on how to use the tool. If you encounter any issues or have any questions when using PACE, please email PLOS at figures@plos.org. Please note that Supporting Information files do not need this step.
